# RANK/RANKL/OPG axis genes relation to cognitive impairment in children with transfusion-dependent thalassemia: a cross-sectional study

**DOI:** 10.1186/s12887-022-03479-9

**Published:** 2022-07-20

**Authors:** Suzan Omar Mousa, Asmaa Hosni Abd El-Hafez, Mostafa Ahmed Abu El-ela, Mohamed Aboul-fotouh Mourad, Rasha Nady Saleh, Samira Zain Sayed

**Affiliations:** 1 Department of Pediatrics, Children’s University hospital, Faculty of Medicine, Minia University, El-Minya, Egypt; 2 Department of Clinical Pathology, Faculty of Medicine, Minia University, El-Minya, Egypt; 3 Department of Radiodiagnosis, Faculty of Medicine, El-Minya, Egypt; 4 Department of Neuropsychiatry, Faculty of Medicine, El-Minya, Egypt

**Keywords:** RANK/RANKL/OPG, Cognition, IQ, Transfusion-dependent thalassemia

## Abstract

**Background:**

RANK/RANKL/OPG axis was implicated in many pathological conditions. The study aimed to assess the relationship between the studied RANK, RANKL, and OPG polymorphisms and alleles and cognitive impairment in children with transfusion-dependent thalassemia (TDT).

**Methods:**

This study included 60 TDT children. Real-time PCR was done for: rs1805034, rs1245811, and rs75404003 polymorphisms for the RANK gene, rs9594782 and rs2277438 polymorphisms for the RANKL gene, and rs207318 polymorphism for the OPG gene. The intelligence quotient (IQ) was assessed using the Wechsler Intelligence Scale for Children-Third Edition.

**Results:**

TDT children had a low average total IQ, verbal IQ, and borderline performance IQ. RANK rs1805034 (C > T) had a significant effect on total IQ (*p* = 0.03). Its TT polymorphism and the CT polymorphism of RANKL rs9494782 (C > T) had a significantly lower total IQ (*p* = 0.01 for both). The G allele of the RANKL rs2277438 (G > A) had a significantly lower total IQ (*p* = 0.02). RANK rs1805034 (C > T) and RANKL rs2277438 (G > A) significantly affected verbal IQ (*p* = 0.01 and 0.03). TT genotype of RANK rs1805034 (C > T) had significantly lower verbal IQ (*p* = 0.002). Furthermore, the GG genotype of RANKL rs2277438 (G > A) had a significantly lower verbal and performance IQ than the AA genotype (*p* = 0.04 and 0.01 respectively), and its G allele had a significantly lower performance IQ than the A allele (*p* = 0.02).

**Conclusion:**

TDT children had low average total and verbal IQ while their performance IQ was borderline. The RANK/RANKL/OPG pathway affects cognition in TDT children, as some of the studied genes’ polymorphisms and alleles had significant effects on total, verbal, and performance IQ of the studied TDT children.

## Background

Beta thalassemia syndromes are mostly autosomal recessive disorders characterized by beta-globin chains synthesis genetic deficiency. More than 200 mutations cause thalassemia [1]. Beta thalassemia spectrum varies from severe transfusion-dependent anemia in the homozygous state to mild to moderate non-transfusion-dependent microcytic hypochromic anemia in the heterozygous state [2]. Excess unpaired alpha-globin chains aggregate and damage red cell membranes, leading to premature destruction of erythroid precursors resulting in ineffective erythropoiesis. These events cause anemia with erythroid hyperplasia and extramedullary hematopoiesis [3].

Transfusion-dependent thalassemia (TDT) causes several health problems due to profound anemia and frequent blood transfusions such as splenomegaly, bone disease, growth delay, endocrinal disturbances, and blood transfusion-transmitted infections [4, 5]. In addition to long-term transfusion therapy, complications related to iron overload, such as iron overload cardiomyopathy, account for most deaths in thalassemia patients [6].

Regular blood transfusion and chelation therapy have increased the life expectancy in thalassemia patients. Neurological involvement, as a result, has become more evident with the advancement in the age of thalassemia patients [7, 8]. Although most were subclinical, a broad spectrum of neurological complications has also been reported, such as cognitive impairment and cerebrovascular diseases [9].

Multiple risk factors contributing to cognitive impairment in TDT include anemia, iron overload, chronic hypoxia, asymptomatic brain infarcts, and visual and auditory toxicity of deferoxamine [10–14]. Although there is some controversy on the relation between brain iron overload and cognitive impairment in thalassemia, for instance, Manara et al. in 2019 found no evidence of iron overload in brain tissue except in the choroid plexuses. They concluded that iron overload might not directly cause cognitive impairment in thalassemia. However, they proposed that choroid plexus’ iron overload may cause cognitive impairment indirectly. As neurodegeneration secondary to choroid plexus iron overload produces free radicals in the cerebrospinal fluid or tissues contiguous to regions strictly related to cognition [15].

Tumor necrosis factor (TNF) is a pro-inflammatory cytokine that controls the expression of numerous signaling pathways implicated in the progression of immunological reactions related to the development of various vascular and metabolic diseases [16]. Osteoprotegerin (OPG) is a cytokine of the TNF receptor superfamily.

The receptor activator of nuclear factor-κB (RANK) and RANK ligand (RANKL) are a receptor-ligand pair of the TNF receptor superfamily. The RANK/RANKL/OPG axis has emerged as the critical molecular pathway in bone metabolism [17].

Previous studies have elucidated the crosstalk between endothelial cells and osteoblasts during osteogenesis, thus connecting angiogenesis with osteogenesis. However, the cellular mechanisms involved are mainly unknown, but growing evidence suggests that the RANK/RANKL/OPG triad may play a significant role in vascular calcification and different disease mechanisms. Many studies confirmed the critical role of the RANK/RANKL/OPG axis in pathological angiogenesis and inflammation, in addition to its role in cell survival through vascular endothelial growth and activation of the nuclear factor kappa light chain enhancer of activated B cells (NF-κB) pathway [18].

It had been documented that RANK/RANKL/OPG axis signaling is implicated in CNS functioning and corresponding pathologies, processes of differentiation, and cell death. It was reported that this axis is involved in the differentiation of cells involved in neuroinflammation, predominantly in microglia and in resident macrophages and inflammatory cells migrating across the blood-brain barrier. They act as neuroprotectants after brain damage [19].

This study aimed to assess the relationship between the studied RANK, RANKL, and OPG polymorphisms and alleles and cognitive impairment in children with TDT.

## Subjects and methods

### Study design and participants

This cross-sectional study was carried out at the Pediatric department, Minia University Children and Maternity Hospital, Faculty of Medicine, Minia University, from September 2019 till May 2021. It included 60 children already diagnosed with transfusion-dependent thalassemia, based on previous hemoglobin electrophoresis and clinical course.

They were recruited from the pediatric hematology outpatient clinic and pediatric hematology in-patient ward. All patients were on a regular blood transfusion program every 2–6 weeks and on deferasirox iron chelation therapy for at least 12 months before participating in the study. Age ranged between 5 and 16 years, and there was no sex predilection. Iron overload in the studied children was assessed by serum ferritin, and liver and cardiac MRI.

Children with mental disorders, history of cerebrovascular accidents, any chronic disease other than TDT, or refused to participate were excluded from the study.

### Data collection

#### Baseline clinical assessment

All included children were subjected to detailed medical history taking and thorough clinical examination with particular emphasis on the history of the age of the first transfusion, transfusion burden/year (ml/kg/year), and history of splenectomy, the average frequency of transfusion, and type and duration of chelation therapy. In addition, the socioeconomic status score was determined for every participant child according to El-Gilany et al. [20], a modification of the old scoring system of Fahmy and El-Sherbini [21].

#### Liver and cardiac MRI

Liver iron concentration (LIC) in mg/g dry weight and T2* MRI was performed in the Department of Radiology, Minia University Children and Maternity Hospital, using MR Philips ingenia 1.5 Tesla (Philips Medical Systems, Netherlands), as part of regular follow-up of the patients.

#### Laboratory analysis

The following laboratory investigations were done for all participants: CBC, serum ferritin, liver function tests.

About 6 ml of venous blood were withdrawn from each subject by sterile venipuncture, 2 ml were collected on two sterile vacutainers containing EDTA solutions tubes, this tube was used for CBC assay by an automated cell counter (CelltacES, Nihon Kohden, Germany). The remaining 4 ml were put on serum separator gel tubes then were allowed to clot for 30 minutes at 37 °C before centrifugation for 15 minutes at 3500 rpm. The expressed serum measured serum ferritin using fully automated clinical chemistry auto-analyzer system Konelab 60i (Thermo Electron Incorporation, Finland). The remaining serum was stored at − 20 °C.

#### Molecular analysis

Real-time PCR was done for the following SNPs: rs1805034, rs1245811, and rs75404003 polymorphisms for the RANK gene, rs9594782 and rs2277438 polymorphisms for the RANKL gene, and rs207318 polymorphism for the OPG gene. It was carried on DT lite 4 Real-Time PCR System (DNA Technology, Russian).

For IQ assessment, all the patients were subjected to IQ assessment by using the Arabic version of the Wechsler Intelligence Scale for Children-Third Edition (WISC-R) [22]. This test assesses children’s intelligence on three scales: total IQ, verbal IQ, and performance IQ. Total-scale IQ is based on ten tests incorporated in the verbal and performance (non-verbal) IQ scales. The administration time was approximately 60–90 minutes.

Verbal IQ is based on information, similarities, arithmetic, comprehension, and digit span. The comprehension subtest is a scale of the student’s social knowledge and the depth of development of morals. Similarities subtest measures logic, abstract thinking, and verbal reasoning, while information is a scale of general knowledge, education, and long-term memory of his experience. Arithmetic and digit span subtests are measures of working, short, and long-term memory. Performance IQ is based on picture completion, coding, picture arrangement, block design, and object assembly. Block design measures analyzing and synthesizing an abstract design and producing the design from colored plastic. Picture completion subtest measures students’ capability of recognizing closely related items. The Object Assembly subtest is a measure of the ability of visualization of item parts of Mazes. The mazes subtest measures perceptual organization, visual-motor coordination, and self-control. The IQ was graded based on the following guidelines: ≥ 130 very superior, 129–120 superior, 119–110 high average, 109–90 average, 89–80 low average, 79–70 borderline, ≤69 extremely low [23].

### Statistical data analysis

Data will be coded, entered, and analyzed using SPSS (statistical package for social sciences) version 20. Descriptive statistics were calculated and expressed as mean ± standard deviation (SD) for quantitative data and as number and percent for qualitative data. Analytical statistics were done by using independent sample t-test (comparison of quantitative data between two groups) and by ANOVA and post-hoc test (comparison of quantitative data among more than two groups). Correlation testing was done by using Pearson’s and Spearman’s correlation coefficients. Binary logistic regression and linear regression analyses were performed to detect the associated independent factors affecting IQ and test for confounding factors’ effect on IQ. *p*-value < 0.05 was considered significant.

## Results

In this study, 38 (63.3%) of the studied TDT children were males, their mean age was 13 ± 4.1 years, and 32 (53.3%) of them were a result of a consanguineous marriage. Regarding their socioeconomic status, 26 (43.3%) were considered as very-low socioeconomic status, 20 (33.3%) were of low socioeconomic status, and 14 (23.3%) were of medium socioeconomic status. Furthermore, their mean age of starting blood transfusion was 19.2 ± 9 months, 34 (57%) of them were splenectomized, their mean BMI was 18.3 ± 2.1, and their mean age of starting chelation was 7 ± 4.2 years. Furthermore, their mean pre-transfusion hemoglobin was 5.7 ± 0.54 g%. In addition, their mean serum ferritin was 4282.6 ± 2635 ng/ml, their mean liver iron concentration was 12.2 ± 7.7 mg/g dry weight, and their mean cardiac T2* MRI was 17.1 ± 6.3 ms.

**Fig. 1:**
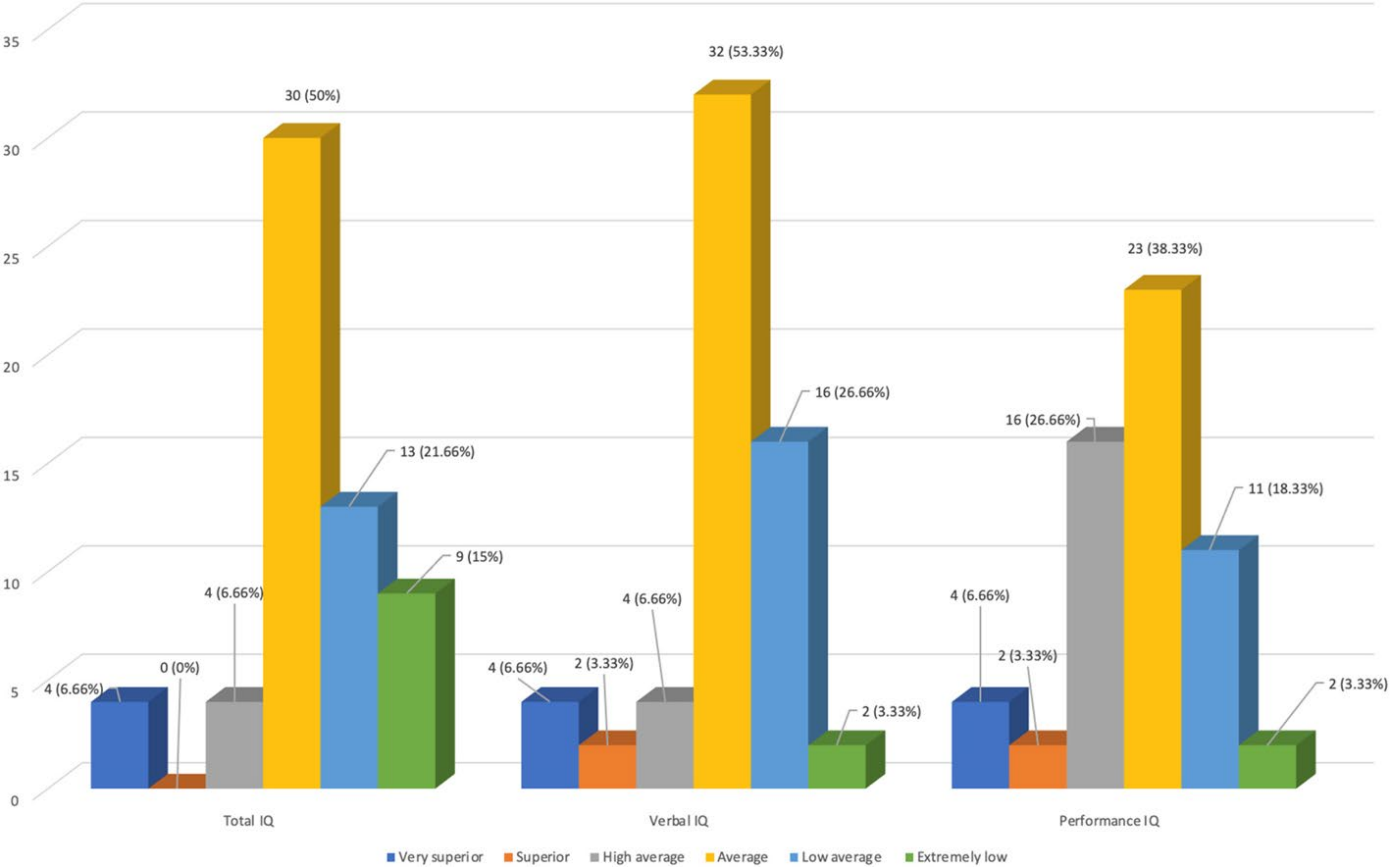
Frequency of different total, verbal, and performance IQ categories among studied TDT children

Their mean total IQ was 80 ± 11.5 (low average), and their mean verbal IQ was 83. 7 ± 14.1 (low average), while their mean performance IQ was 77.7 ± 9.4 (borderline). The frequencies of different total, verbal, and performance IQ categories are shown in Fig. 1.

**Fig. 2:**
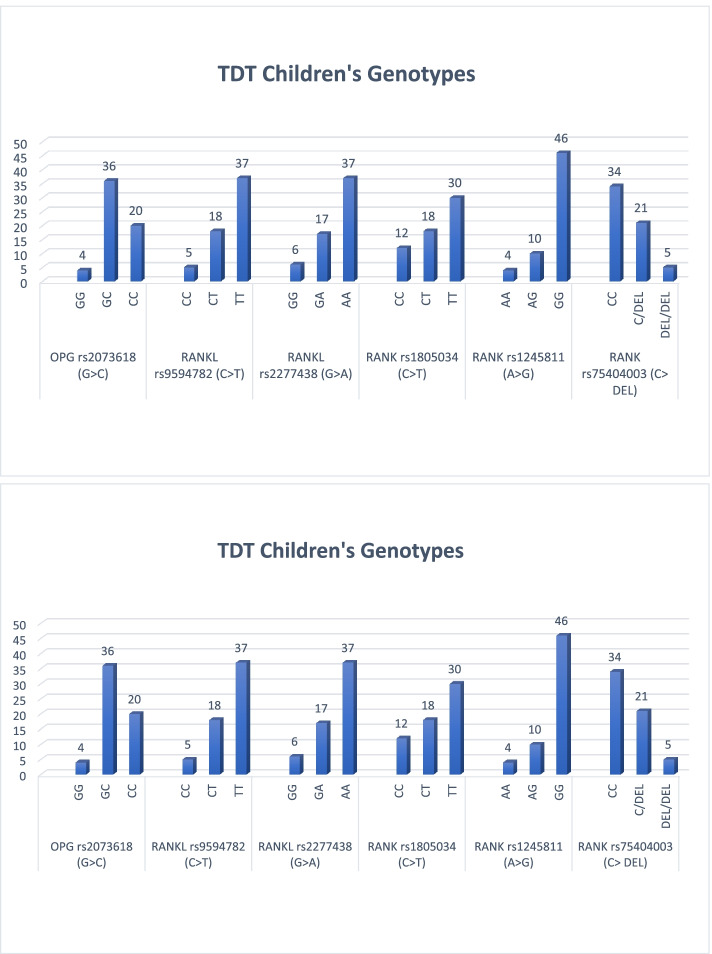
Frequency of the studied polymorphisms genotypes among TDT children

Frequency distribution of different genotypes of the studied polymorphisms among the TDT children are shown in Fig. 2.

Regarding the relation of the studied genes polymorphisms with total IQ, RANK rs1805034 (C > T) polymorphisms had a significant relation with total IQ (*p* = 0.03). Moreover, the post hoc test revealed that TDT children with its TT polymorphism had significantly lower total IQ than children with the CT polymorphism (*p* = 0.01). While RANKL rs9494782 (C > T) polymorphisms did not have a significant relation with total IQ (*p* = 0.1), however, children having the CT polymorphism of this gene had significantly lower total IQ than children with the CC polymorphism on performing the post hoc test (*p* = 0.01). On studying the alleles, children with the G allele of the RANKL rs2277438 (G > A) had significantly lower total IQ (*p* = 0.02), with a significant unstandardized β coefficient of − 5.09 (CI 95%: − 9.8 – − 3.37) (*p* = 0.02) (Table 1).

**Table 1:** The relation of the studied polymorphisms and alleles of the RANK/RANKL/OPG axis with the total IQ of TDT children

Gene		TDT total IQ			
		mean ± SD	p	unstandardized coefficient β (95% CI)	p
**OPG rs2073618 (G > C)**					
**Polymorphisms**	GG	76.5 ± 11	0.2	0.84 (−4.33–6)	0.75
	GC	80 ± 11			
	CC	80 ± 13			
**Alleles**	C	80 ± 11.8	0.84	−0.5 (−4.8–3.7)	0.8
	G	79.5 ± 10.5			
**RANK rs1805034 (C > T)**					
**Polymorphisms**	CC	79.5 ± 3	0.03*	−2.4 (− 6.14–1.4)	0.2
	CT	85.4 ± 13			
	TT	76.8 ± 11.5			
**Alleles**	C	82 ± 9	0.14	−3.2 (−7.4–1.1)	0.14
	T	78.8 ± 12			
**RANK rs1245811 (A > G)**					
**Polymorphisms**	AA	82.5 ± 17	0.6	0.55 (−4.5–5.6)	0.8
	AG	77 ± 12			
	GG	80.4 ± 11			
**Alleles**	A	79.3 ± 13.5	0.8	0.74 (−5–6.5)	0.8
	G	80 ± 11			
**RANK rs75404003 (C > DEL)**					
**Polymorphisms**	CC	81 ± 11	0.3	−2.8 (−7.3–2)	0.2
	C DEL	80 ± 12.5			
	DEL DEL	73 ± 5.5			
**Alleles**	C	80.7 ± 11.3	0.21	−3 (−7.6–1.7)	0.2
	Del	77.7 ± 11			
**RANKL rs9494782 (C > T)**					
**Polymorphisms**	CC	87 ± 19	0.1	- 0.2 (−4.8–4.45)	0.95
	CT	76 ± 12			
	TT	80.4 ± 9.5			
**Alleles**	C	80 ± 15	0.94	−0.18 (−5.05–4.7)	0.94
	T	80 ± 11.3			
**RANKL rs2277438 (G > A)**					
**Polymorphisms**	GG	73 ± 5	0.2	4.15 (−0.14–8.44)	0.1
	GA	78 ± 10			
	AA	82 ± 12			
**Alleles**	A	81 ± 11.8	0.02*	−5.09 (−9.8 – −3.37)	0.02*
	G	76 ± 8.5			

* Statistical significance *p* < 0.05

Regarding the relation of the studied genes polymorphisms with verbal IQ, polymorphisms RANK rs1805034 (C > T) and RANKL rs2277438 (G > A) had significant relation with verbal IQ (*p* = 0.01 and 0.03). At the same time, the homomutant TT genotype of RANK rs1805034 (C > T) had significantly lower verbal IQ than the CT hetero-mutant genotype (*p* = 0.002), while the GG genotype of RANKL rs2277438 (G > A) had a significantly lower verbal IQ than the AA homomutant genotype (*p* = 0.04). The unstandardized β coefficient of RANKL rs2277438 (G > A) polymorphisms was 6.5 (CI 95%: 1.4–11.6) (*p* = 0.02). However, no alleles showed a significant statistical relation with verbal IQ (Table 2).

**Table 2:** The relation of the studied polymorphisms and alleles of the RANK/RANKL/OPG axis with the verbal IQ of TDT children

Gene		TDT verbal IQ			
		mean ± SD	p	unstandardized coefficient β (95% CI)	p
**OPG rs2073618 (G > C)**					
**Polymorphisms**	GG	80.5 ± 12	0.7	−0 .95-(−7.3–5.4)	0.8
	GC	85 ± 14			
	CC	82 ± 14			
**Alleles**	C	83.4 ± 14	0.8	0.7(−4.55–5.9)	0.8
	G	84 ± 13.5			
**RANK rs1805034 (C > T)**					
**Polymorphisms**	CC	83 ± 7	0.01*	−3.7- (−8.3–0.8)	0.1
	CT	92 ± 16			
	TT	79 ± 13			
**Alleles**	C	87 ± 12	0.1	−4.98-(−10.2–0.2)	0.1
	T	82 ± 14.4			
**RANK rs1245811 (A > G)**					
**Polymorphisms**	AA	86.5 ± 17	0.8	0.08 (− 6.1–6.3)	1
	AG	81 ± 12			
	GG	84 ± 14.3			
**Alleles**	A	83.5 ± 13.4	1	0.13 (−6.9–7.2)	1
	G	83.6 ± 14			
**RANK rs75404003 (C > DEL)**					
**Polymorphisms**	CC	83.5 ± 12	0.3	−1.7 (−7.3–4)	0.55
	C DEL	85 ± 17			
	DEL DEL	75 ± 11			
**Alleles**	C	84 ± 13	0.53	−1.8 (− 7.55–3.9)	0.5
	Del	82.3 ± 15.6			
**RANKL rs9494782 (C > T)**					
**Polymorphisms**	CC	92 ± 26.3	0.3	−0.735 (−6.4–5)	0.8
	CT	80 ± 15			
	TT	84 ± 11			
**Alleles**	C	84.3 ± 19.4	0.8	−0.85 (−7.55–3.9)	0.8
	T	83.4 ± 11.7			
**RANKL rs2277438 (G > A)**					
**Polymorphisms**	GG	74.5 ± 4.5	0.03*	6.5 (1.4–11.6)	0.02*
	GA	80 ± 11			
	AA	87 ± 15			
**Alleles**	A	85.5 ± 14.5	**0.1**	−7.97 (−6.8–5.)	0.1
	G	77.6 ± 9			

* Statistical significance *p* < 0.05

No polymorphism showed a significant relation to the performance IQ. However, the GG genotype of RANKL rs2277438 (G > A) had a significantly lower performance IQ than the AA genotype (*p* = 0.01). Furthermore, the G allele of the RANKL rs2277438 (G > A) had significantly lower performance IQ than the A allele (*p* = 0.02), with a significant unstandardized β coefficient − 3.924 (− 7.789 – − 2.318) (*p* = 0.02) (Table 3).

**Table 3:** The relation of the studied polymorphisms and alleles of the RANK/RANKL/OPG axis with the performance IQ of TDT children

Gene		TDT performance IQ			
		mean ± SD	p	unstandardized coefficient β (95% CI)	p
**OPG rs2073618 (G > C)**					
**Polymorphisms**	GG	75 ± 9	0.65	12 (−2.2–6.25)	0.35
	GC	77 ± 9			
	CC	79 ± 10.4			
**Alleles**	C	78 ± 9.6	0.43	−1.4 -(−4.95–2)	0.4
	G	76.8 ± 8.7			
**RANK rs1805034 (C > T)**					
**Polymorphisms**	CC	77.5 ± 3.5	0.3	−1.2- (−4.3–1.9)	0.43
	CT	80.4 ± 10.			
	TT	76 ± 10			
**Alleles**	C	78.7 ± 7	0.4	−1.6-(−5.15–1.9)	0.4
	T	77 ± 10			
**RANK rs1245811 (A > G)**					
**Polymorphisms**	AA	80 ± 15	0. 6	0.3 (−3.9–4.4)	0.9
	AG	75 ± 10			
	GG	78 ± 9			
**Alleles**	A	77 ± 12	0.9	0.4 (−4.35–5.1)	0.9
	G	77.7 ± 8.7			
**RANK rs75404003 (C > DEL)**					
**Polymorphisms**	CC	79 ± 10	0.35	−2.7- (−6.4–1)	0.15
	C DEL	76.3 ± 8			
	DEL DEL	74 ± 5			
**Alleles**	C	78.4 ± 9.8	0.13	−2.9-(−6.75–0.9)	0.13
	Del	75.5 ± 7			
**RANKL rs9494782 (C > T)**					
**Polymorphisms**	CC	83 ± 12	0.12	0.45 (−3.4–4.2)	0.84
	CT	74.3 ± 10			
	TT	79 ± 8			
**Alleles**	C	77 ± 11	0.8	0.45 (−3.5–4.4)	0.8
	T	77.8 ± 8.7			
**RANKL rs2277438 (G > A)**					
**Polymorphisms**	GG	70 ± 8	0.12	3.2 (−0.3–6.7)	0.14
	GA	78 ± 10			
	AA	79 ± 9			
**Alleles**	A	78.6 ± 9	0.02*	−3.9- (−7.8--0.06-)	0.02*
	G	74.7 ± 9.5			

* Statistical significance *p* < 0.05

RANK rs1805034 (C > T) polymorphism showed significant negative correlation with total and verbal IQ, as (*r* = − 0.33, *p* = 0.01) and (*r* = − 0.33, *p* = 0.009) respectively. While RANKL rs2277438 (G > A) polymorphism showed significant positive correlation with verbal IQ (*r* = 0.38, *p* = 0.01). Although its G allele correlation with performance IQ did not reach statistical significance (*p* > 0.05) (Table 4).

**Table 4:** Correlations of factors affecting total, verbal, and performance IQ in TDT children

	Total IQ		Verbal IQ		Performance IQ	
	r	p	r	p	r	p
**RANK rs1805034 (C > T)**	−0.33	0.01*	−0.33	0.009*	–	–
**RANKL rs2277438 (G > A)**	–	–	0.38	0.01*	–	–
**G allele of RANKL rs2277438 (G > A)**	0.22	0.79	–	–	0.25	0.05
**Age (years)**	−0.54	0.001*	− 0.63	0.001*	− 0.32	0.01*
**Sex**	0.08	0.5	0.21	0.09	0.1	0.4
**Socioeconomic level**	0.18	0.15	0.2	0.06	0.17	0.17
**BMI**	0.113	0.3	0.12	0.3	−0.09	0.4
**Pre-transfusion Hb (gm%)**	0.09	0.4	0.09	0.4	0.04	0.7
**Serum ferritin (ng/ml)**	−0.32	0.01*	−0.33	0.009*	−0.28	0.02*
**LIC (mg/g dry weight)**	−0.019	0.8	0.13	0.3	0.02	0.8

*Hb* hemoglobin, *BMI* body mass index, *LIC* liver iron concentration.

* Statistical significance *p* < 0.05

Age and serum ferritin had significant negative correlations with total, verbal, and performance IQ in this study regarding other factors that might affect TDT children’s IQ (Table 4).

Linear regression analyses of the factors significantly correlating with total and verbal IQ are shown in Table 5. RANK rs1805034 (C > T) polymorphism’s standardized 𝛃 coefficient reached statistical significance for predicting changes in total and verbal IQ (*p* = 0.009 and 0.03 respectively). Whereas RANKL rs2277438 (G > A) polymorphism’s standardized 𝛃 coefficient was borderline significant for predicting changes in verbal IQ (*p* = 0.06) (Table 5).

**Table 5:** Multiple linear regression analysis of factors correlating with total and verbal IQ

	Total IQ			Verbal IQ		
	Standardized coefficient (𝛃)	t	p	Standardized coefficient (𝛃)	t	p
**RANK rs1805034 (C > T)**	− 0.17	−1.67	0.009*	− 0.21	− 2.2	0.03*
**RANKL rs2277438 (G > A)**	–		–	0.1	0.39	0.06
**Age (years)**	−0.51	−4.83	0.001*	−0.58	−5.7	0.001*
**Serum ferritin (ng/ml)**	−0.23	−2.23	0.03*	−0.22	− 2.2	0.02*
**R**^**2**^	R^2^ = 0.4 (F = 11.8; df = 3)			R^2^ = 0.5 (F = 13.9; df = 4)		

* Statistical significance *p* < 0.05

## Discussion

Neurological involvement, such as cognitive impairment and cerebrovascular diseases, has become more evident with improved patient care and increased life expectancy in TDT [7–9].

Our study demonstrated that TDT children had low total, verbal, and performance IQ. This deterioration of IQ scores is compatible with Meymandi et al. and Canatan et al. studies. They reported significantly lower verbal IQ subsets, performance IQ subsets, and academic problems in 60% of thalassemia children [24, 25]. Our results also agreed with the Egyptian study done by Raafat et al. in 2015, who found that TDT patients had marked lower performances and full-Scale IQ scores [26]. Cognitive impairment in thalassemia has several risk factors, such as chronic anemia resulting in chronic hypoxia, bone marrow expansion and may be iron overload. These factors cause the production of free radicals in the cerebrospinal fluid and tissues contiguous to regions strictly related to cognition [12, 14, 15, 27].. Moreover, asymptomatic brain infarcts, pain and discomfort related to treatment complications, mood changes, and frequent absences from school may aggravate cognitive impairment [10, 11, 28, 29].

Contradictory to our results, Economou et al., 2006 and Alzaree et al., 2018 reported that TDT children had higher scores on the verbal scale [27, 30]. However, other studies claimed that intelligence decline does not necessarily occur in TDT children, and they are just slightly lower than their healthy counterparts. They attributed that to little caring about the quality of education of those children [24, 31]. The contradictory results can be explained by using different assessment tools, and the extent to which the illness had affected the body and how these patients are supported may differ among different study groups [32, 33].

Our study found that polymorphisms of RANK rs1805034 (C > T) affected total IQ and verbal IQ. Moreover, TDT children with TT polymorphism had significantly lower total and verbal IQ than children with CT polymorphism. Additionally, children with the CT polymorphism of RANKL rs9494782 (C > T) had significantly lower total IQ than those with the CC polymorphism. RANKL rs2277438 (G > A) was significantly affecting verbal IQ. Its AA homomutant form had a significantly higher verbal and performance IQ than the GG genotype. Children with the G allele of the RANKL rs2277438 (G > A) had significantly lower total and performance IQ.

Unfortunately, limited research is available addressing the relation of SNPs in our study with cognition; however, these SNPs had been linked to other pathological conditions. Previous studies found that RANK rs1805034 (C > T) polymorphisms might be involved in cardiovascular disorders, and its minor C allele was protective for diastolic dysfunction and osteoporotic hip fracture [16, 34, 35]. The RANKL SNP rs2277438 has been reported as a factor that contributes to the radiographic progression of Rheumatoid arthritis in the Japanese population [36]. Also, Rhee et al., and Cho et al., showed that SNP srs2277438 and rs9594782 of the RANKL gene influenced vascular calcification and bone metabolism in humans [37, 38].

A meta-analysis was done by Song et al. showed that rs2073618 G > C(1181G > C) polymorphisms of the OPG gene were closely related to cardiovascular disorders [39]. In this study, the GG polymorphism of this SNP had lower total, verbal and performance IQ, but this did not reach statistical significance.

A recent study by Ping-Hsun et al. found a relationship between cognitive impairment and the RANK/RANKL/OPG axis; they reported that serum RANKL levels were positively correlated to the cognitive function tests in hemodialysis patients [40]. Moreover, another study found that enhancing RANKL/RANK signaling in animals by recombinant RANKL significantly reduced ischemic brain infarct volume [41].

Also, serum OPG levels were significantly related to cognition [42], and the OPG SNP T245G was significantly associated with an increased risk of ischemic brain stroke [43].

The effect the RANK/RANKL/OPG axis has on cognition may be attributed to its effect on the circulating endothelial progenitor cells, which play a crucial role in pathological angiogenesis and inflammation [18, 44]. As Moazzami et al. in 2020 reported, a lower number of endothelial progenitor cells is associated with cognitive impairment and impairment of verbal memory functions [45]. In addition to the axis involvement in the differentiation of cells involved in neuroinflammation, predominantly in microglia, and in resident macrophages and inflammatory cells migrating across the blood-brain barrier [19].

## Limitations

This study is a single-center study that needs to be incorporated into a multi-center study to determine the results on a broader scale with larger sample size. In addition, other genes involved in RANK/RANKL/OPG pathway should also be studied in the future concerning their effect on cognition in transfusion-dependent thalassemia patients. Another limitation is that we have not compared our results with healthy children or children with non-transfusion-dependent thalassemia of the same age and sex. Nevertheless, the aim of this study was limited to assessing the studied genetic markers in TDT children.

## Conclusion

In conclusion, TDT children in this study had low average total and verbal IQ while their performance IQ was borderline. Furthermore, this study showed that RANK rs1805034 affected total and verbal IQ, CT polymorphism of RANKL rs9494782 was associated with lower total IQ, and RANKL rs2277438 affected verbal IQ, and its GG genotype was associated with lower performance IQ. Moreover, the RANKL rs2277438 G allele was associated with lower total and performance IQ. Therefore, the RANK/RANKL/OPG pathway impacts cognition in TDT children, and the above SNPs act as genetic markers for cognition impairment in TDT children.

## Data Availability

The datasets analyzed during the current study available from the corresponding author on reasonable request.

## Abbreviations

TDT: Transfusion-dependent thalassemia
RANK: Receptor activator of nuclear factor-κB
RANKL: Receptor activator of nuclear factor-κB ligand
OPG: Osteoprotegerin
SNP: Single-nucleotide polymorphism
IQ: Intelligence quotient
